# Functional neuroimaging in the acute phase of Takotsubo syndrome: volumetric and functional changes of the right insular cortex

**DOI:** 10.1007/s00392-020-01602-3

**Published:** 2020-01-30

**Authors:** Wolfgang Dichtl, Noora Tuovinen, Fabian Barbieri, Agne Adukauskaite, Thomas Senoner, Andrea Rubatscher, Florian Hintringer, Christian Siedentopf, Axel Bauer, Elke R. Gizewski, Ruth Steiger

**Affiliations:** 1 University Hospital for Internal Medicine III (Cardiology and Angiology), Medical University of Innsbruck, Innsbruck, Austria; 2 Department of Neuroradiology, Medical University of Innsbruck, Innsbruck, Austria; 3 Neuroimaging Research Core Facility, Medical University of Innsbruck, Innsbruck, Austria

**Keywords:** Brain–heart axis, Limbic system, Insular cortex, Takotsubo syndrome, Resting-state fMRI

## Abstract

**Background:**

A brain–heart interaction has been proposed in Takotsubo syndrome (TTS). Structural changes in the limbic system and hypoconnectivity between certain brain areas in the chronic phase of the disease have been reported, but little is known concerning functional neuroimaging in the acute phase. We hypothesized anatomical and functional changes in the central nervous system and investigated whole-brain volumetric and functional connectivity alterations in the acute phase TTS patients compared to controls.

**Methods:**

Anatomical and resting-state functional magnetic resonance imaging were performed in postmenopausal females: thirteen in the acute TTS phase and thirteen healthy controls without evidence of coronary artery disease. Voxel-based morphometry and graph theoretical analysis were applied to identify anatomical and functional differences between patients and controls.

**Results:**

Significantly lower gray matter volumes were found in TTS patients in the right middle frontal gyrus (*p* = 0.004) and right subcallosal cortex (*p* = 0.009) compared to healthy controls. When lower threshold was applied, volumetric changes were noted in the right insular cortex (*p* = 0.0113), the right paracingulate cortex (*p* = 0.012), left amygdala (*p* = 0.018), left central opercular cortex (*p* = 0.017), right (*p* = 0.013) and left thalamus (*p* = 0.017), and left cerebral cortex (*p* = 0.017). Graph analysis revealed significantly (*p* < 0.01) lower functional connectivity in TTS patients compared to healthy controls, particularly in the connections originating from the right insular cortex, temporal lobes, and precuneus.

**Conclusion:**

In the acute phase of TTS volumetric changes in frontal regions and the central autonomic network (i.e. insula, anterior cingulate cortex, and amygdala) were noted. In particular, the right insula, associated with sympathetic autonomic tone, had both volumetric and functional changes.

**Graphic abstract:**

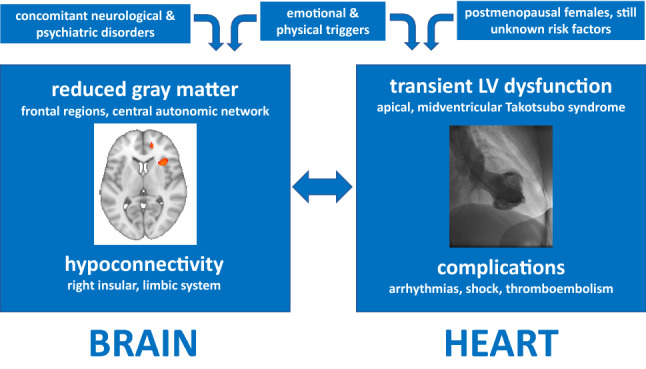

## Introduction

Many cardiac and neurological disorders influence each other via a still poorly understood “brain–heart axis”. Stroke or epilepsy involving certain brain areas, such as the right insular cortex, has been reported to be associated with cardiac arrhythmias and/or Takotsubo syndrome (TTS) [1, 2].

The insular cortex, considered the “hidden fifth lobe”, is situated at the base of the Sylvian fissure. Grossly, it consists of the anterior and posterior insula, bisected by the central insular sulcus. The insula integrates autonomic, motor, and sensory functions through its reciprocal connections with other parts of the brain, notably the limbic system [3]. The right insula seems to exhibit a sympathetic dominance; in fact, its stimulation induces heart rate increase and/or pressor response; on the contrary, the stimulation of the left insula leads to parasympathetic tone increase with heart rate decrease and/or depressor responses. Pressor responses are seen with rostral insular cortex stimulation, and depressor effects with caudal insular stimulation. The insula has a major role in cardiovascular regulation, specifically in limbic-autonomic regulation [4].

Only few studies with limited patient samples have been performed using functional brain imaging during the acute episode of TTS. Resting-state functional magnetic resonance imaging (rs-fMRI) provides information on functional connectivity, i.e. communication between different areas of the brain. Rs-fMRI allows to study which brain regions have related spontaneous fluctuations of the blood oxygen level-dependent signal. Specific regions form networks that associate with different functional domains [5]. Graph theoretical modeling can describe functional connectivity as nodes (regions) and edges (connections) that can reflect changes in the topological architecture [6].

A previous single-photon emission computed tomography (SPECT) study on TTS patients showed significant cerebral blood flow increase in the hippocampus, brainstem and basal ganglia and decrease in the prefrontal cortex in the acute phase of three patients. These changes gradually subsided, but were still present after 4–6 weeks. The authors concluded that recovery of left ventricular dysfunction precedes that of brain activation [7]. An autonomic challenge task-fMRI study found significant differences in the pattern of activation of the insular cortex, amygdala and the right hippocampus in four patients who had experienced TTS in the past compared to eight healthy controls [8].

Furthermore, an rs-fMRI study on default mode network found increase in the precuneus and decrease in the ventromedial prefrontal cortex connectivity in non-acute phase TTS patients compared to healthy controls [9]. Another group applied whole-brain and subnetwork analyses for parasympathetic network, sympathetic network and default mode network in 15 non-acute phase TTS patients and found significant connectivity decrease in all networks, but in particular, in brain regions associated with autonomic functions and regulation of limbic system (i.e. amygdala, hippocampus, cingulate gyrus) [10].

Few studies applied voxel-based morphometry (VBM) in TTS patients. Such a previous VBM study on 20 TTS patients showed reduced grey matter (GM) volume in left and right amygdala and at the right amygdala–hippocampus border. Furthermore, there was decreased cortical thickness in the antero-ventral insulae. In the same study cohort, structural connectivity analysis showed reduction mainly within the limbic system, whereas the subnetwork analysis of autonomic nervous system showed decrease in the left amygdala, both hippocampi, left parahippocampus and right putamen [11].

So far, no previous fMRI-studies have investigated TTS patients in the acute phase of the disease. We set out to analyse volumetric differences with VBM and functional connectivity differences with graph methods in TTS patients in the acute phase compared to healthy controls. Possible anatomical and functional changes in these patients may provide a deeper understanding of the role of the interaction between the cardiovascular and central nervous system in TTS.

## Methods

### Patients’ characteristics

Postmenopausal females were recruited at the department of Internal Medicine III, Medical University of Innsbruck: 13 healthy controls (aged 65.6 ± 7.7 years) and 13 patients with an acute TTS episode (aged 71.3 ± 6.2 years) with symptom onset less than 24 h before emergency coronary angiography. Diagnosis of TTC was performed according to criteria described by international consensus documents and/or large registries [12–14]. Exclusion criteria were impossibility to perform brain MRI within the next 72 h, limited capability to communicate in German, and contraindications to perform a MRI examination (e.g. implantation of a cardiac device, mechanical heart valves, claustrophobia, severe obesity). None of the TTS patients had a previous diagnosis of depression or anxiety-related disorders. Triggers for the TTS were either emotional (*n* = 6), physical (*n* = 4) or not detectable (*n* = 3). Apical ballooning was found in ten TTS patients whereas a midventricular type was found in three TTS patients. TTS patients presented with clinical features mimicking acute myocardial infarction. Therefore, all coronary angiographies were performed without any delay to exclude the need for urgent revascularization. Brain MRI was performed on the second or third day after hospital admission in clinically stable patients free of pulmonary congestion and/or sustained new-onset arrhythmias. Healthy controls did not suffer from overt neurological or psychiatric diseases or any relevant medical conditions except for arterial hypertension (*n* = 8) and/or hyperlipidemia (*n* = 7). Detailed characteristics of the study cohort are shown in Table 1.

**Table 1:** Values are mean ± standard deviation, *n* (%) or median (interquartile range)

	TTS (*n* = 13)	Controls (*n* = 13)
Age, years	71.3 ± 6.2	65.6 ± 7.7
Systolic blood pressure, mmHg	124 ± 29	144 ± 14
Heart rate, beats/min	84.5 ± 15.7	67.5 ± 7.6
Body mass index	24.5 ± 3.2	24.8 ± 2.8
Serum creatinine, mg/dl	0.81 ± 0.16	0.96 ± 0.31
Clinical features of heart failure/myocardial damage		
Left ventricular ejection fraction, %	51.0 ± 12.8	58.4 ± 4.6
N-terminal pro-B-type natriuretic peptide, ng/l	3066 ± 3737	161 ± 142
Creatine kinase, U/l	239 ± 164	85 ± 36
Troponin T, ng/l	646.9 ± 503.5	6.5 ± 2.9
Medical history		
Arterial hypertension	8 (61.5)	8 (61.5)
Diabetes mellitus	1 (7.7)	0 (0)
Hyperlipidemia	8 (61.5)	7 (53.8)
Chronic obstructive pulmonary disease	1 (7.7)	0 (0)
Peripheral artery disease	1 (7.7)	0 (0)

The study procedures were performed according to the Declaration of Helsinki and approved by the ethics committee of the Medical University of Innsbruck. All subjects signed informed consent form prior to inclusion in the study. The study was registered at ClinicalTrials.gov (NCT02240056).

### MRI data acquisition

Structural and functional sequences were acquired with a 3 T whole-body MRI scanner (Magnetom Verio, Siemens, Germany). T1 magnetization prepared rapid acquisition gradient echo (MPRAGE) sequence parameters were set to repetition time = 1950 ms, echo time = 3.30 ms, slice thickness = 1.0 mm, flip angle = 9° and field of view = 220 × 178.75 mm^2^. Rs-fMRI sequence (single-shot gradient echo planar imaging) parameters were set to TR = 2.40 s, TE = 30 ms, flip angle = 90°, field of view = 1540 × 1540 mm^2^, slice thickness = 2.5 mm and acquisition time of 7 min. Subjects were told to lay still and relaxed with their eyes closed during the acquisition.

### Voxel-based morphometry

To investigate possible volumetric differences, T1-weighted images were processed with an optimized VBM protocol [15] with FMRIB Software Library (FSL) tools [16]. Brain-extracted (BET) T1 images were segmented into white matter (WM), GM and cerebrospinal fluid (CSF) volume probability maps with FMRIB’s automated segmentation tool (FAST). To avoid bias during the registration process, a symmetric study-specific GM template was created from all of the participants’ images by registering in the Montreal Neurological Institute (MNI) 152 space with the affine registration tool FLIRT. After nonlinear registration with FNIRT, the resulting images were averaged to create the template. After registration onto the GM template, the optimized protocol involved a compensation for the local contraction/enlargement caused by the nonlinear component of the transformation: each voxel of each registered GM image was multiplied by the Jacobian of the warp field which corrects for the differences in brain volume. The images were then smoothed with an isotropic Gaussian kernel of 3 mm. Randomise tool was used (alpha < 0.01/0.02, 5000 permutations, threshold-free cluster enhancement (TFCE), age as covariate) to identify group differences between patients and controls.

### Graph theoretical analysis

Graph theoretical analysis was applied to investigate whole-brain functional connectivity. FSL’s MELODIC ICA was used for rs-fMRI preprocessing: 6 mm smoothing, high-pass temporal filtering (100 s), and FLIRT and FNIRT registration of functional images through T1 image (6 degrees of freedom) to MNI template (12 degrees of freedom), and motion correction. Mean signals of WM, CSF (respiration and pulse effects) and six motion parameters were regressed from the preprocessed functional data [17, 18]. Then brain parcellation of 384 regions was created. Atlas of intrinsic connectivity of homotopic areas (AICHA) [19] was selected as it provides a parcellation that takes into account the intrinsic connectivity of homotopic areas. A graph that consisted of 384 nodes was created. To obtain 384 × 384 graph matrices, functional time series of each parcellated region were averaged and Pearson correlated with the other regions. Then obtained correlation matrices were Fisher-transformed according to *z* = ln((1 + *r*)/(1 − *r*))/2 with Matlab. Network-based statistics [20] was used to perform *t* test (alpha < 0.01, false discovery rate (FDR)-corrected, 100,000 permutations) between patients and controls on every edge in the connectivity matrices.

## Results

### Volumetric differences

**Fig. 1:**
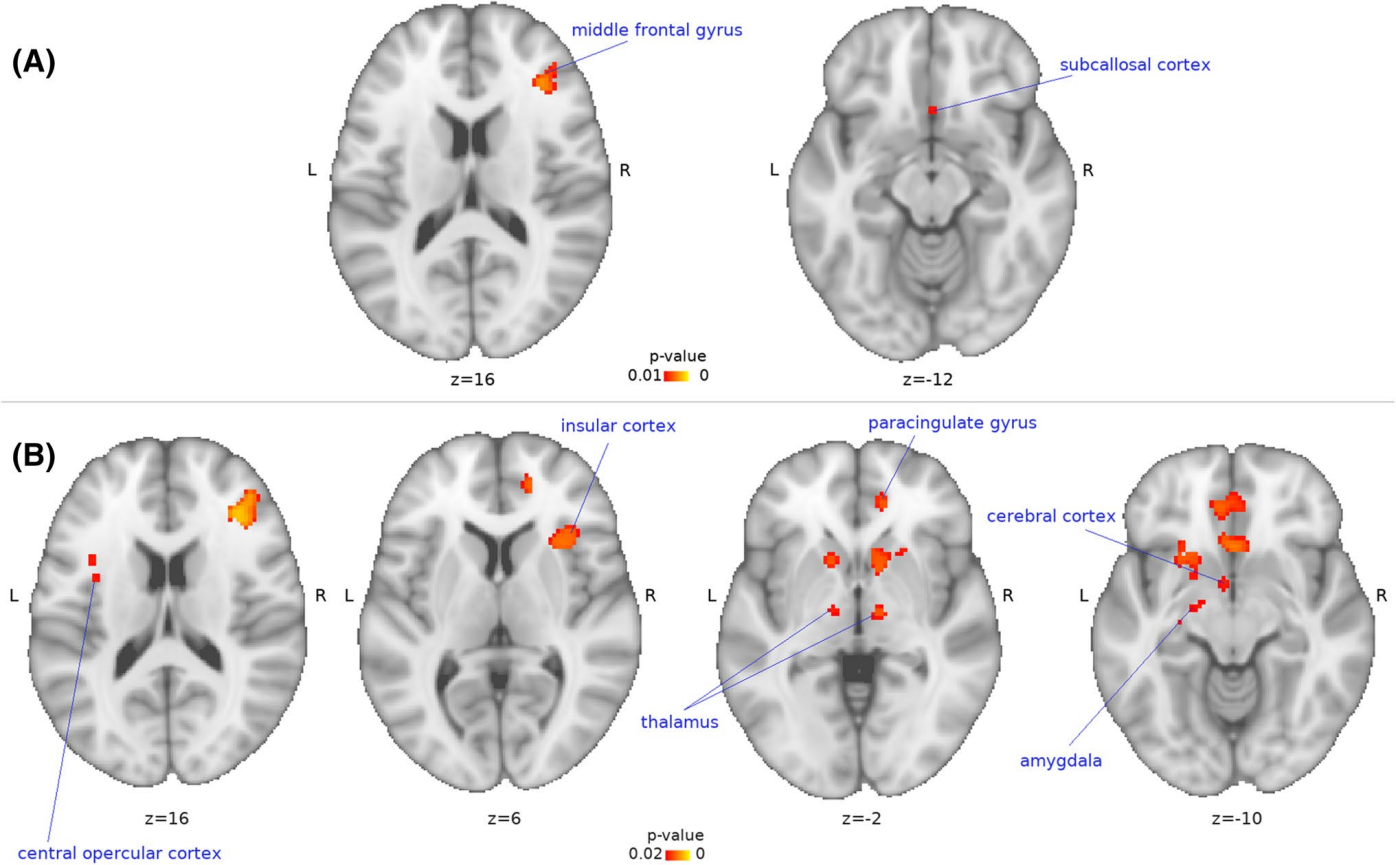
TTS patients had lower (in orange) grey matter volume (voxel-based morphometry (VBM), randomise, 5000 permutations, TFCE) compared to controls

To study volumetric differences of GM in patients compared to controls, VBM analysis was applied (Fig. 1). TTS patients had significantly lower (in orange) grey matter volume in right middle frontal gyrus (*x* = 36, *y* = 32, *z* = 16, *p* = 0.004) which extended to right insula (*x* = 30, *y* = 19, *z* = 4, *p* = 0.0113), left central opercular cortex (*x* = − 34, *y* = 2, *z* = 14, *p* = 0.017), right paracingulate gyrus (*x* = 16, *y* = 46, *z* = 4, *p* = 0.012), right (*x* = 10, *y* = − 12, *z* = − 2, *p* = 0.013) and left thalamus (*x* = − 10, *y* = − 12, *z* = − 4, *p* = 0.017), left cerebral cortex (*x* = − 4, *y* = 2, *z* = − 10, *p* = 0.017), left amygdala (*x* = − 16, *y* = − 10, *z* = − 10, *p* = 0.018), and right subcallosal cortex (*x* = 2, *y* = 18, *z* = − 12, *p* = 0.009) compared to controls. Clusters coordinates for voxels with the maximum statistical significance are given, and results are presented in Montreal Neurological Institute (MNI) coordinates and overlaid on template brain.

### Functional connectivity differences

**Fig. 2:**
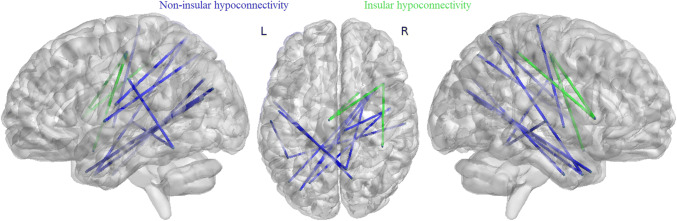
TTS patients had differences in functional connectivity compared to controls among 384 regions from AICHA-atlas (insular connections are shown in green, non-insular connections are shown in blue)

Graph theoretical methods were applied to identify possible functional connectivity differences between 384 brain regions in TTS patients compared to controls (Fig. 2). TTS patients had significantly (*p* < 0.01, 100.000 permutations, *t* test FDR-corrected) lower (in green) functional connectivity in following insular connections compared to controls: right intraparietal sulcus 1 (*x* = 40, *y* = − 40, *z* = 51) and right anterior insula gyrus 4 (*x* = 41, *y* = 15, *z* = 4) (Tstat: 4.80); right anterior insula gyrus 1 (*x* = 19, *y* = 7, *z* = − 19) and left paracentral lobule gyrus 1 (*x* = − 7, *y* = − 17, *z* = 51) (Tstat: 5.49); right anterior insula gyrus 4 and left paracentral lobule gyrus 1 (Tstat: 4.62). Other regions with significantly lower (in blue) connectivity in TTS patients were left intraoccipital sulcus 1 (*x* = − 24, *y* = − 72, *z* = 32) and right inferior temporal gyrus 1 (*x* = 45, *y* = − 7, *z* = − 36) (Tstat: 5.30), left middle occipital gyrus 1 (*x* = − 32, *y* = − 78, *z* = 25) and right middle temporal pole gyrus 2 (*x* = 35, *y* = 12, *z* = − 34) (Tstat: 4.62), left middle occipital gyrus 3 (*x* = − 42, *y* = − 71, *z* = 18) and right middle temporal pole gyrus 2 (Tstat: 4.09), left middle occipital gyrus 1 and right middle temporal pole gyrus 3 (*x* = 26, *y* = 6, *z* = − 36) (Tstat: 5.82), right superior temporal gyrus 2 (*x* = 47, *y* = − 7, *z* = − 2) and right paracentral lobule gyrus 3 (*x* = 7, *y* = − 26, *z* = 70) (Tstat: 5.67), right superior temporal gyrus 4 (*x* = 60, *y* = − 20, *z* = 2) and left fusiform gyrus 4 (*x* = − 43, *y* = − 50, *z* = − 17) (Tstat: 5.28), left Rolandic operculum gyrus 2 (*x* = − 51, *y* = − 9, *z* = 14) and right precuneus gyrus 6 (*x* = 8, *y* = − 60, *z* = 62) (Tstat: 5.82), right middle temporal pole 3 and right precuneus gyrus 6 (Tstat: 5.62), left superior temporal gyrus 1 (*x* = − 55, *y* = − 1, *z* = 2) and right precuneus gyrus 9 (*x* = 13, *y* = − 68, *z* = 49) (Tstat: 5.00), and left postcentral sulcus 1 (*x* = − 58, *y* = − 18, *z* = 32) and left fusiform gyrus 4 (Tstat: 5.24). Results are presented in Montreal Neurological Institute (MNI) coordinates and overlaid on template brain. Nodes are presented in their centroid stereotaxic coordinates.

## Discussion

Our study presents functional neuroimaging in the acute phase of TTS. Compared to healthy controls, we found lower GM volumes and lower functional connectivity in acute postmenopausal TTS patients.

Volumetric differences in TTS patients were found in the right middle frontal gyrus and right subcallosal cortex compared to healthy controls. When lower threshold was applied, volumetric changes were noted in the right insular cortex, right paracingulate cortex, left amygdala, left central opercular cortex, right and left thalamus and left cerebral cortex.

Furthermore, TTS patients showed a lower functional connectivity, particularly in connections from the right anterior insular cortex, temporal lobes, and right precuneus compared with controls.

There were both volumetric and functional differences in the right insula in TTS patients during the acute phase. Our study confirms previously found reduced GM volume in the amygdala and the right insular cortex [11], as well as volumetric changes in the right paracingulate cortex. These regions form the central autonomic network (CAN) which regulates the cardiovascular system [4]. In particular, the right insular cortex is linked to the amygdala and plays an important role in the autonomic control of cardiac activity, and was previously linked to cardiac arrhythmias and TTS [21–23]. Right insular strokes often lead to a cardiac autonomic derangement, complex arrhythmias [21] and peculiar electrocardiographic changes [23] which are also found in TTS [24]. Insular damage was one of the predominant features in stroke patients who suffered from concomitant TTS [22]. In fact, involvement of the insular cortex during stroke results in autonomic dysregulation and adverse cardiac outcomes [25].

Interestingly, we found lower connectivity of the right insular cortex, whose functional changes were previously suggested in task-fMRI study, where differences in the pattern of activation of the insular cortex, amygdala and the right hippocampus were noted in TTS patients compared to controls [8]. Previous functional connectivity studies in the chronic phase of TTS (months to years after the acute event) found significant decreases [10] as well as increases [22] in a network that involved the left anterior insula. Our findings, however, suggest a damage to the right insula causing variations to the resting-state functional connectivity.

As generally shown for anxiety-related disorders, the insular–amygdala connectivity may additionally play an important role in TTS, which finally leads to sympathetic hyperactivity and baroreflex impairment [26, 27, 28]. Although we have not found differences in connectivity to amygdala, insular subdivisions (i.e. dorsal anterior, ventral anterior, posterior) are further connected with different functional networks (e.g. executive control, somatomotor and default mode network) [29], and a damage to this region can have wider effects on network communication. In fact, our connectivity analyses revealed that part of the insular cortex (Rolandic operculum) had lower connectivity with the default mode network (i.e. the right precuneus), which further had lower connectivity with temporal pole in TTS patients compared to controls.

Apart from the right insular cortex, frontal brain regions may play a key role in TTS as well. We found volumetric differences in TTS patients in the right middle frontal gyrus compared to controls. The frontal cortex is critically involved in the control of fear, aggression, mating behaviour and other aspects of emotional processing via connections to the amygdala and other limbic structures [30–33]. Our results are in line with a SPECT study that showed significantly decreased cerebral blood flow in the prefrontal cortex in the acute phase of TTS [7].

Furthermore, the lower functional connectivity in TTS patients in temporal regions was previously noted also in the chronic phase, where decrease in functional [10] and structural [11] connectivity was found in a network that involved these regions as part of the parasympathetic network, as well as decrease in cingulate as part of the sympathetic and limbic networks.

In conclusion, our findings support the concept that neurological and/or psychiatric disorders are extremely common in TTS patients, as originally described in a landmark publication of Templin and co-workers from the InterTAK Registry [2]. Anatomical changes in the right insula, paracingulate cortex and amygdala influence the cardiovascular system, and there are functional disruptions of the right insula in the acute phase of TTS. Therefore, brain areas associated with sympathetic autonomic tone seem to be involved in the pathogenesis of TTS. Neurological diseases involving the insular cortex should prompt physicians to screen for TTS and/or consider continuous cardiac monitoring in the early phase of clinical presentation.

## References

[1] Porto I, Della Bona R, Leo A, Proietti M, Pieroni M, Caltagirone C (2013). Stress cardiomyopathy (takotsubo) triggered by nervous system diseases: a systematic review of the reported cases. Int J Cardiol.

[2] Templin C, Ghadri J, Bataiosu DR, Dieckmann J, Jaguszewski M, Sarcon A (2015). Clinical characteristics, diagnosis and outcome of Takotsubo cardiomyopathy—results from the International Takotsubo Registry (InterTAK Registry). N Engl J Med.

[3] Ay H, Koroshetz WJ, Benner T, Vangel MG, Melinosky C, Arsava EM (2006). Neuroanatomic correlates of stroke-related myocardial injury. Neurology.

[4] Nagai M, Dote K, Kato M, Sasaki S, Oda N, Kagawa E (2017). The insular cortex and takotsubo cardiomyopathy. Curr Pharm Des.

[5] Van Dijk KR, Hedden T, Venkataraman A, Evans KC, Lazar SW, Buckner RL (2010). Intrinsic functional connectivity as a tool for human connections: theory, properties, and optimization. J Neurophysiol.

[6] Rubinov M, Sporns O (2010). Complex network measures of brain connectivity: uses and interpretations. Neuroimage.

[7] Suzuki H, Matsumoto Y, Kaneta T, Sugimura K, Takahashi J, Fukumoto Y (2013). Evidence for brain activation in patients with takotsubo cardiomyopathy. Circ J.

[8] Pereira VH, Marques P, Magalhães R, Português J, Calvo L, Cerqueira JJ (2016). Central autonomic nervous system response to autonomic challenges is altered in patients with a previous episode of Takotsubo cardiomyopathy. Eur Heart J Acute Cardiovasc Care.

[9] Sabisz A, Treder N, Fijalkowska M, Sieminski J, Fijalkowska P, Naumczyk R (2016). Brain resting state functional magnetic resonance imaging in patients with takotsubo cardiomyopathy an inseparable pair of brain and heart. Int J Cardiol.

[10] Templin C, Hänggi J, Klein C, Topka MS, Hiestand T, Levinson RA (2019). Altered limbic and autonomic processing supports brain-heart axis in Takotsubo syndrome. Eur Heart J.

[11] Hiestand T, Hänggi J, Klein C, Topka MS, Jaguszewski M, Ghadri JR (2018). Takotsubo syndrome associated with structural brain alterations of the limbic system. J Am Coll Cardiol.

[12] Ghadri JR, Wittstein IS, Prasad A, Sharkey S, Dote K, Akashi YJ (2018). International expert consensus document on takotsubo syndrome (part I): clinical characteristics, diagnostic criteria, and pathophysiology. Eur Heart J.

[13] Ghadri JR, Wittstein IS, Prasad A, Sharkey S, Dote K, Akashi YJ (2018). International expert consensus document on takotsubo syndrome (part II): diagnostic workup, outcome, and management. Eur Heart J.

[14] Stiermaier T, Santoro F, Graf T, Guastafierro F, Tarantino N, De Gennaro L (2018). Prognostic value of N-terminal pro-B-type natriuretic peptide in takotsubo syndrome. Clin Res Cardiol.

[15] Good CD, Johnsrude IS, Ashburner J, Henson RN, Friston KJ, Frackowiak RS (2001). A voxel-based morphometric study of ageing in 465 normal adult human brains. NeuroImage.

[16] Jenkinson M, Beckmann CF, Behrens TE, Woolrich MW, Smith SM (2012). FSL. NeuroImage.

[17] Dagli MS, Ingeholm JE, Haxby JV (1999). Localization of cardiac-induced signal change in fMRI. NeuroImage.

[18] Windischberger C, Langenberger H, Sycha T (2002). On the origin of respiratory artifacts in BOLD-EPI of the human brain. Magn Reson Imaging.

[19] Joliot M, Jobard G, Naveau M, Delcroix N, Petit L, Zago L (2015). AICHA: an atlas of intrinsic connectivity of homotopic areas. J Neurosci Methods.

[20] Zalesky A, Fornito A, Bullmore ET (2010). Network-based statistic: identifying differences in brain networks. NeuroImage.

[21] Colivicchi F, Bassi A, Santini M, Caltagirone C (2004). Cardiac autonomic derangement and arrhythmias in right-sided stroke with insular involvement. Stroke.

[22] Yoshimura S, Toyoda K, Ohara T, Nagasawa H, Ohtani N, Kuwashiro T (2008). Takotsubo cardiomyopathy in acute ischemic stroke. Ann Neurol.

[23] Pasquini M, Laurent C, Kroumova M, Masse I, Deplanque D, Leclerc X (2006). Insular infarcts and electrocardiographic changes at admission: results of the prognostic of insular cerebral infarcts study (PRINCESS). J Neurol.

[24] Gassanov N, Le MT, Caglayan E, Hellmich M, Erdmann E, Er F (2018). Novel ECG-based scoring tool for prediction of takotsubo syndrome. Clin Res Cardiol.

[25] Oppenheimer S (2006). Cerebrogenic cardiac arrhythmias: cortical lateralization and clinical significance. Clin Auton Res.

[26] Silva AR, Magalhães R, Arantes C, Moreira PS, Rodrigues M, Marques P (2019). Brain functional connectivity is altered in patients with Takotsubo syndrome. Sci Rep.

[27] Baur V, Hänggi J, Langer N, Jäncke L (2013). Resting-state functional and structural connectivity within an insula-amygdala route specifically index state and trait anxiety. Biol Psychiatry.

[28] Vaccaro A, Despas F, Delmas C, Lairez O, Lambert E, Lambert G (2014). Direct evidences for sympathetic hyperactivity and baroreflex impairment in Tako Tsubo cardiopathy. PLoS ONE ONE.

[29] Liu X, Chen X, Zheng W, Xia M, Han Y, Song H (2018). Altered functional connectivity of insular subregions in Alzheimer's disease. Front Aging Neurosci.

[30] Klein C, Hiestand T, Ghadri JR, Templin C, Jäncke L, Hänggi J (2017). Takotsubo syndrome—predictable from brain imaging data. Sci Rep.

[31] Thayer JF, Lane RD (2009). Claude Bernard and the heart-brain connection: further elaboration of a model of neurovisceral integration. Neurosci Biobehav Rev.

[32] Wager TD, Waugh CE, Linquist M, Noll DC, Fredrickson BL, Taylor SF (2009). Brain mediators of cardiovascular responses to social threat: part I: reciprocal dorsal and ventral sub-regions of the medial prefrontal cortex and heart-rate reactivity. Neuroimage.

[33] Olson CR, Colby CL, Kandel ER, Schwartz JH, Jessell TM (2013). The organization of cognition. Principles of neural science.

